# Distribution of D-amino acids in vinegars and involvement of lactic acid bacteria in the production of D-amino acids

**DOI:** 10.1186/2193-1801-2-691

**Published:** 2013-12-27

**Authors:** Yuta Mutaguchi, Taketo Ohmori, Hirofumi Akano, Katsumi Doi, Toshihisa Ohshima

**Affiliations:** Department of Biomedical Engineering, Faculty of Engineering, Osaka Institute of Technology, 5-16-1 Omiya, Asahi-ku, Osaka, 535-8585 Japan; Central Research Institute of Mizkan Group Corporation, 2-6 Nakamura-cho, Handa, 475-0873 Japan; Microbial Genetic Division Institute of Genetic Resources Faculty of Agriculture, Kyushu University, 6-10-1 Hakozaki, Higashi-ku, Fukuoka, 812-8581 Japan

**Keywords:** D-Amino acid, Vinegar, Lactic acid bacteria, Fermentation, UPLC

## Abstract

Levels of free D-amino acids were compared in 11 vinegars produced from different sources or through different manufacturing processes. To analyze the D- and L-amino acids, the enantiomers were initially converted into diastereomers using pre-column derivatization with *o*-phthaldialdehyde plus *N*-acethyl-L-cysteine or *N*-*tert*-butyloxycarbonyl-L-cysteine. This was followed by separation of the resultant fluorescent isoindol derivatives on an octadecylsilyl stationary phase using ultra-performance liquid chromatography. The analyses showed that the total D-amino acid level in lactic fermented tomato vinegar was very high. Furthermore, analysis of the amino acids in tomato juice samples collected after alcoholic, lactic and acetic fermentation during the production of lactic fermented tomato vinegar showed clearly that lactic fermentation is responsible for the D-amino acids production; marked increases in D-amino acids were seen during lactic fermentation, but not during alcoholic or acetic fermentation. This suggests lactic acid bacteria have a greater ability to produce D-amino acids than yeast or acetic acid bacteria.

## Background

The α-amino acids that serve as building blocks for proteins each possess α-carbon bearing a hydrogen atom, an amino group, a carboxyl group and one of 20 different side chains. With the exception of glycine, the α-amino acids are optically active, and two optical isomers (L- and D-) of each amino acid can be formed. Recently, developed analytical techniques have shown that there are D-amino acids in various foods including wine (Kato et al., 2011), milk (Rubio-Barroso et al., 2006), beer (Erbe and Brückner, 2000), vegetable and fruit (Brückner and Westhauser, 2003). In particular, fermented foods are known to contain several D-amino acids, including D-alanine (D-Ala), D-aspartate (D-Asp) and D-glutamate (D-Glu) (Brückner and Hausch, 1989; Abe et al., 1999; Jin et al., 1999). These D-amino acids are thought to be derived from the starting materials used to prepare those foods, or they are produced during microbial fermentation (Brückner et al., 1993). Given the presence of D-amino acids in many kinds of foods, it follows that their effects on taste of foods have been focused. It is known that D-Ala, D-leucine (D-Leu) and D-phenylalanine (D-Phe) are all sweeter than their L-amino acid counterparts (Solms et al., 1965; Schiffman et al., 1981; Kawai et al., 2012), suggesting D-amino acids may be contribute to the taste quality of fermented foods. Okada et al. showed that D-Ala actually contributes increase of umami taste of sake, which is one kind of fermented food (Okada et al., 2013). Thus, in fermented foods, a better understanding of the distribution of D-amino acids and their production mechanisms during fermentation helps clarify their effects on the taste and might enable to improve the taste by the control of their productions.

Vinegar, which is produced through the fermentation of grains, vegetables and fruits, is a popular flavoring used widely all over the world. Yeasts and acetic acid bacteria are mainly responsible for the fermentation processes that produce vinegar. Yeasts convert sugar to alcohol in a process known as the alcoholic fermentation, after which, acetic acid bacteria such as the *Acetobacter* species convert the alcohol to acetic acid in acetic fermentation. When grains are used as the starting materials for vinegars, starch is converted to sugars by “*Koji*” fungi such as the *Aspergillus* species before alcoholic fermentation (Machida et al., 2008). Moreover, the Central Research Institute of Mizkan Group Corporation (Handa, Japan) has experimentally produced vinegar using a procedure that includes lactic fermentation (details are provided in the Methods).

With that as background, we hypothesized that by examining distributions of D-amino acids in vinegars through different fermentation processes, we could gain information about the mechanisms involved in the production of D-amino acids during the fermentation process. In the present study, we determined the levels of D-amino acids in 11 vinegars produced from several different sources and through several different manufacturing processes, and then investigated which fermentation processes participate in the production of D-amino acids.

## Results and discussion

### Measurement of D-amino acids in vinegars

We first determined the concentrations of 16 types of D-amino acid in various vinegars (Table 1) and calculated their relative percentages using the formula 100 × D/(D + L), where D is the concentration of the D-form and D + L is the total concentration of each amino acid (Tables 2 and 3). The relative standard deviations (n = 3) of the absolute amounts were below 10% in all samples. The presence of D-amino acids was confirmed in all the vinegars tested; however, the concentrations of D-Asp and D-Glu detected in apple vinegar were too low to be accurately measured (data not shown).

**Table 1:** **The vinegars used for the** **D** **-amino acid analyses**

Material	Vinegar
Grain vinegar	Rice vinegar
	Nonglutinous brown rice vinegar
	Nonglutinous brown rice black vinegar
	High-brix nonglutinous brown rice black vinegar
	High-brix nonglutinous brown rice black vinegar matured in barrel
Fruit vinegar	Apple vinegar
	High-brix apple vinegar
	White wine vinegar
	Balsámico
Vegetable vinegar	Tomato vinegar
	Lactic fermented tomato vinegar

**Table 2:** **Determination of free** **D** **-amino acids in grain vinegar samples**

Amino acid	Rice vinegar		Nonglutinous brown rice vinegar		Nonglutinous brown rice black vinegar		High-brix nonglutinous brown rice black vinegar		High-brix nonglutinous brown rice black vinegar matured in barrel	
	μM^a^	% **D** ^b^	μM	% **D**	μM	% **D**	μM	% **D**	μM	% **D**
D-Asp	7.2	2.7	62.3	3.2	48	2.5	97.3	3.4	631.3	20.5
D-Ser	− ^c^	−	30.6	1.6	18.9	1.0	64.5	2.1	431.4	16.1
D-Gln	−	−	−	−	−	−	−	−	−	−
D-His	−	−	−	−	−	−	−	−	−	−
D-Thr	−	−	−	−	−	−	−	−	−	−
D-Arg	−	−	N. D. ^d^	N. D.	N. D.	N. D.	15.7	0.6	93.2	5.4
D-Ala	15.0	2.3	118.9	3.7	85.1	2.5	203.8	4.2	534.4	9.2
D-Tyr	−	−	−	−	−	−	−	−	148.3	8.7
D-Val	−	−	−	−	−	−	N. D.	N. D.	6.3	0.2
D-Met	−	−	−	−	−	−	−	−	−	−
D-Trp	−	−	−	−	−	−	−	−	−	−
D-Phe	−	−	8.2	0.6	9.3	0.6	29.5	1.2	214.9	11.4
D-*allo*-Ile	−	−	−	−	−	−	−	−	−	−
D-Leu	−	−	N. D.	N. D.	N. D.	N. D.	12.6	0.3	224.7	6.7
D-Glu	N. D.	N. D.	40.3	1.9	31.7	1.4	62.0	2.3	241.6	16.6
D-Asn	−	−	17.7	1.7	10.2	0.9	52.0	2.8	188.9	17.1

^a^Concentrations cited are smaller one among the amino acid concentrations determined using NAC and NBC as chiral regents.

^b^Relative percentages of D-amino acids were calculated as 100 × [D-amino acid]/[D-amino acid + L-amino acid].

^c^−, not detected.

^d^N. D., not determined. The D-amino acid peak was confirmed, but the concentration of the D-amino acid was too low to be determined.

**Table 3:** **Determination of free** **D** **-amino acids in fruit and vegetable vinegar samples**

Amino acid	High-brix apple vinegar		Balsámico		White wine vinegar		Tomato vinegar		Lactic fermented tomato vinegar	
	μM^a^	% **D** ^b^	μM	% **D**	μM	% **D**	μM	% **D**	μM	% **D**
D-Asp	113.4	3.5	17.4	5.7	N. D. ^d^	N. D.	69.0	0.4	660.9	2.3
D-Ser	− ^c^	−	N. D.	N. D.	N. D.	N. D.	−	−	50.5	1.4
D-Gln	−	−	−	−	−	−	−	−	−	−
D-His	−	−	−	−	−	−	−	−	−	−
D-Thr	−	−	−	−	−	−	−	−	−	−
D-Arg	−	−	10.7	0.7	N. D.	N. D.	−	−	53.2	4.2
D-Ala	149.6	13.4	34.4	3.4	10.1	3.3	41.1	0.3	1,355.7	6.5
D-Tyr	−	−	−	−	−	−	−	−	−	−
D-Val	−	−	N. D.	N. D.	N. D.	N. D.	−	−	134.3	8.7
D-Met	−	−	−	−	−	−	−	−	29.3	78.1
D-Trp	−	−	−	−	−	−	−	−	−	−
D-Phe	N. D.	N. D.	N. D.	N. D.	−	−	−	−	96.3	2.7
D-*aIlo*-Ile	N. D.	N. D.	−	−	−	−	−	−	521.8	35.8
D-Leu	−	−	N. D.	N. D.	N. D.	N. D.	N. D.	N. D.	401.2	35.1
D-Glu	9.1	2.0	7.0	2.4	N. D.	N. D.	64.7	0.2	265.6	0.7
D-Asn	247.9	13.1	−	−	−	−	62.9	0.5	205.4	1.2

^a^Concentrations cited are smaller one among the amino acid concentrations determined using NAC and NBC as chiral regents.

^b^Relative percentages of D-amino acids were calculated as 100 × [D-amino acid]/[D-amino acid + L-amino acid].

^c^−, not detected.

^d^N. D., not determined. The D-amino acid peak was confirmed, but the concentration of the D-amino acid was too low to be determined.

The levels of D-amino acids in rice vinegars are summarized in Table 2. The total D-amino acid concentrations in rice vinegar, nonglutinous brown rice vinegar, nonglutinous brown rice black vinegar, high-brix nonglutinous brown rice black vinegar and high-brix nonglutinous brown rice black vinegar matured in barrel were 22.2, 278.0, 203.2, 537.4 and 2,715.0 μM, respectively. Thus, among the five grain vinegars tested, the high-brix nonglutinous brown rice black vinegar matured in barrel contained the highest total D-amino acids concentration, by far. This suggests that maturation of the vinegar is pivotal for the D-amino acid production. Consistent with that idea, a positive correlation between maturation time and D-amino acid content was reported for balsámico (Erbe and Brückner, 1998), and it was suggested that the maturation-related increase in D-amino acids resulted from a microbial enzymatic isomerization rather than an entirely acid-catalyzed mechanism. For high-brix nonglutinous brown rice black vinegar matured in barrel, microorganisms from the source vinegar or the barrel may be responsible for the production of the D-amino acids, as is the case with balsámico.

In addition, the total D-amino acid concentrations in high-brix apple vinegar, white wine vinegar, balsámico, tomato vinegar and lactic fermented tomato vinegar were 520.0, 10.1, 69.5, 237.7 and 3,773.2 μM, respectively (Table 3). Among the 11 types of vinegar tested in this study, the highest total D-amino acid concentration was detected in lactic fermented tomato vinegar (3,773.2 μM). Notably, tomato vinegar produced from the same source material but without lactic fermentation showed a much lower total D-amino acid concentration (237.7 μM, 4 kinds). This suggests it is the lactic fermentation that is mainly responsible for the production of D-amino acids.

### D-Amino acid levels during production of lactic fermented tomato vinegar

**Figure 1:**
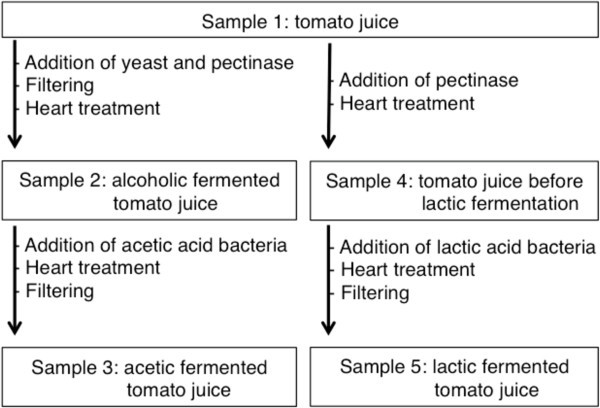
**Samples used assessment of** **D** **-amino acid production during the manufacture of lactic fermented tomato vinegar.** Details of samples are described in the Methods.

**Figure 2:**
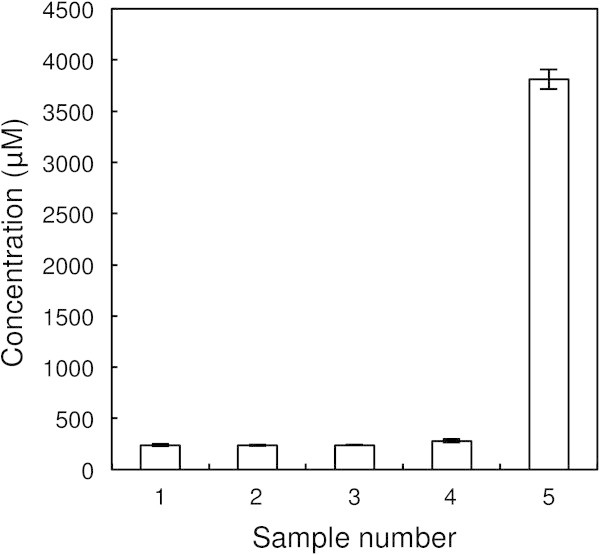
**Total** **D** **-amino acid concentrations produced during the fermentation of lactic fermented tomato vinegar.** The total concentrations (μM) of 16 types of D-amino acid in the samples were calculated as described in the Methods.

Production of lactic fermentation tomato vinegar entails several fermentation steps: alcoholic, acetic or lactic fermentation. To clarify the contribution made by lactic fermentation to the production of D-amino acids in lactic fermented tomato vinegar, we analyzed the D-amino acids present in five samples collected during different fermentation steps (Figure 1). The results are showed in Table 4, and then, based on those results, we tested whether alcoholic, acetic or lactic fermentation is mainly responsible for the D-amino acid production. The total D-amino acid concentrations in the five samples are shown in Figure 2. D-Asp, D-Ala, D-Glu and D-asparagine (D-Asn) were detected in samples 1, 2, 3 and 4, and the respective levels of those four D-amino acids did not greatly differ among the samples. This indicates that the D-amino acids in these samples were derived from the tomato juice, and were not produced by either alcoholic or acetic fermentation. By contrast, sample 5 contained a much higher concentration of D-amino acids than the other four samples. The total concentration of D-amino acids in sample 5 was 12.4-times higher than that in sample 4, and their relative percentage (100 × D/(D + L)) in sample 5 was also much higher (14.3-times) than in sample 4. Furthermore, in the sample 5, D-serine (D-Ser), D-arginine (D-Arg), D-valine (D-Val), D-methionine (D-Met), D-Phe, D-*allo*-isoleucine (D-*allo*-Ile) and D-Leu were detected in addition to D-Asp, D-Ala, D-Glu and D-Asn, and the concentrations of D-Asp, D-Ala and D-Glu in sample 5 were 6.0-, 69.8- and 2.6-times higher than in sample 4, respectively. This suggests that most of the D-amino acids in lactic fermented tomato vinegar are produced during lactic fermentation.

**Table 4:** **Analysis of** D **-amino acids in five samples of lactic fermented tomato vinegar**

Amino acid	Sample 1^a^		Sample 2		Sample 3		Sample 4		Sample 5	
	μM^b^	% **D** ^c^	μM	% **D**	μM	% **D**	μM	% **D**	μM	% **D**
D-Asp	68.2	0.3	68.8	0.4	69.0	0.4	99.4	0.4	599.0	2.7
D-Ser	− ^d^	−	−	−	−	−	−	−	22.7	0.7
D-Gln	−	−	−	−	−	−	−	−	−	−
D-His	−	−	−	−	−	−	−	−	−	−
D-Thr	−	−	−	−	−	−	−	−	−	−
D-Arg	−	−	−	−	−	−	−	−	37.2	65.7
D-Ala	24.6	0.1	29.5	0.2	41.1	0.3	28.1	0.1	1,962.4	10.7
D-Tyr	−	−	−	−	−	−	−	−	−	−
D-Val	−	−	−	−	−	−	−	−	81.6	8.0
D-Met	−	−	−	−	−	−	−	−	32.3	21.6
D-Trp	−	−	−	−	−	−	−	−	−	−
D-Phe	−	−	−	−	−	−	−	−	89.7	3.2
D-*aIlo*-Ile	−	−	−	−	−	−	−	−	420.9	38.6
D-Leu	−	−	−	−	N. D. ^e^	N. D.	−	−	285.0	37.3
D-Glu	67.6	0.2	66.8	0.2	64.7	0.2	81.7	0.2	209.1	0.7
D-Asn	78.0	0.5	71.4	0.6	62.9	0.5	71.7	0.4	71.0	0.5

^a^Details of samples 1–5 are provided in the Methods.

^b^Concentrations cited are smaller one among the amino acid concentrations determined using NAC and NBC as chiral regents.

^c^Relative percentages of D-amino acids were calculated as 100 × [D-amino acid]/[D-amino acid + L-amino acid].

^d^−, not detected.

^e^N. D., not determined. The D-amino acid peak was confirmed, but the concentration of the D-amino acid was too low to be determined.

### D-Amino acids in the culture media conditioned by lactic acid bacteria

**Figure 3:**
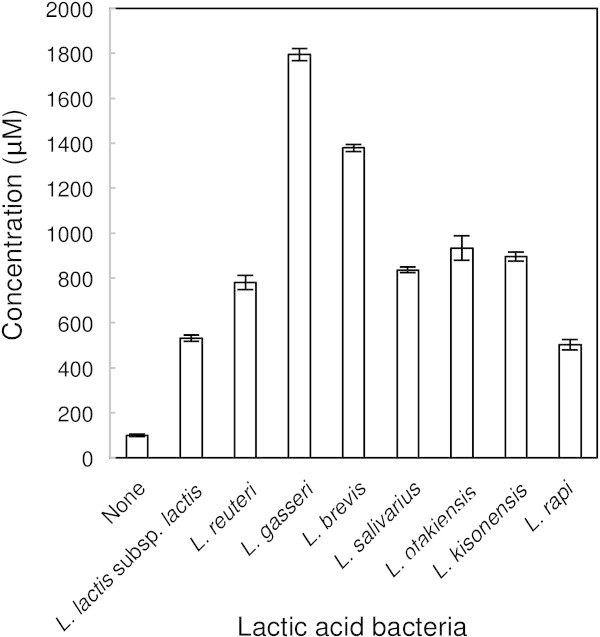
**Total** **D** **-amino acid concentrations in culture medium conditioned by various lactic acid bacteria.** The total concentrations (μM) of 16 types of D-amino acid in the culture medium were calculated as described in the Methods. “None” indicates the total D-amino acid concentration in MRS medium without growth of lactic acid bacteria.

To further confirm the apparent contribution of lactic acid bacteria to the D-amino acids production in lactic fermented tomato vinegar, we next assessed the levels of D-amino acids in the culture medium conditioned by each of eight strains of lactic acid bacteria (Table 5). The results are showed in Table 6, and the total D-amino acid concentrations in the culture media are shown in Figure 3. In each case, the total D-amino acid concentration in the conditioned culture medium was higher than that in the medium without growth of lactic acid bacteria. In particular, marked increases in D-Ala, D-Glu and D-Asp concentrations were observed. The concentrations of several other D-amino acids in the culture medium also increased: D-Ser in medium from all of the eight strains; D-Arg in medium from L*. salivarius*; D-Val in medium from L*. otakiensis*; D-Phe in media from L*. lactis* subsp. *lactis*, L*. gasseri,*L*. brevis* and L*. otakiensis*; D-*allo*-Ile in medium from L*. reuteri* and L*.otakiensis*; D-Leu in media from L*. reuteri* and L*.otakiensis*. This again supports the idea that the high total D-amino acid concentration in lactic fermented tomato vinegar is mainly produced during lactic fermentation.

**Table 5:** **Lactic acid bacteria used for analyzing the** **D** **-amino acids in culture medium**

Strain			Cultivation temperature	Cultivation time
*Lactococcus*	*lactis* subsp*. lactis*	JCM 5805	30°C	72 h
*Lactobacillus*	*reuteri*	JCM 1112	37°C	36 h
	*gasseri*	JCM 1131	37°C	36 h
	*brevis*	JCM 1170	30°C	72 h
	*salivarius*	JCM 1231	37°C	36 h
	*otakiensis*	JCM 15040	30°C	72 h
	*kisonensis*	JCM 15041	30°C	72 h
	*rapi*	JCM 15042	30°C	72 h

**Table 6:** **Analysis of** **D** **-amino acids in culture medium conditioned by lactic acid bacteria**

A										
Amino acid	No lactic acid bacteria		***L. lactis*** subsp. ***lactis*** JCM 5805^a^		***L. reuteri*** JCM 1112		***L. gasseri*** JCM 1131		***L. brevis*** JCM 1170	
	μM^b^	% **D** ^c^	μM	% **D**	μM	% **D**	μM	% **D**	μM	% **D**
D-Asp	32.1	2.7	74.8	6.4	150.2	16.7	225.0	16.3	316.7	24.4
D-Ser	7.7	0.5	13.3	1.1	13.8	1.0	34.9	1.7	20.4	1.1
D-Gln	− ^d^	−	−	−	−	−	−	−	−	−
D-His	−	−	−	−	−	−	−	−	−	−
D-Thr	−	−	−	−	−	−	−	−	−	−
D-Arg	−	−	−	−	−	−	−	−	−	−
D-Ala	33.3	1.1	402.5	13.8	286.0	8.2	1,410.6	41.4	934.4	24.5
D-Tyr	−	−	−	−	−	−	−	−	−	−
D-Val	−	−	−	−	−	−	−	−	−	−
D-Met	−	−	−	−	−	−	−	−	−	−
D-Trp	−	−	−	−	−	−	−	−	−	−
D-Phe	N.D.^e^	−	6.3	0.4	N.D.	−	5.7	0.4	5.5	0.3
D-*aIlo*-Ile	−	−	−	−	41.6	2.9	−	−	−	−
D-Leu	−	−	−	−	31.4	1.2	−	−	N.D.	−
D-Glu	13.3	0.4	22.9	0.8	246.3	8.2	101.9	3.2	88.0	70.7
D-Asn	13.9	1.8	12.6	1.8	11.5	6.3	16.4	5.5	14.2	2.1
**B**										
**Amino acid**			***L. salivarius*** **JCM 1231** ^**a**^		***L. otakiensis*** **JCM 15040**		***L. kisonensis*** **JCM 15041**		***L. rapi*** **JCM 15042**	
			**μM** ^**b**^	**%** **D** ^**c**^	**μM**	**%** **D**	**μM**	**%** **D**	**μM**	**%** **D**
D-Asp			118.3	26.3	128.7	22.0	120.4	22.9	110.1	11.5
D-Ser			13.6	1.0	14.1	1.1	24.0	1.4	12.4	0.9
D-Gln			− ^d^	−	−	−	−	−	−	−
D-His			−	−	−	−	−	−	−	−
D-Thr			−	−	−	−	−	−	−	−
D-Arg			35.9	3.7	−	−	−	−	−	−
D-Ala			537.8	19.0	336.0	9.9	576.2	16.2	309.6	8.7
D-Tyr			−	−	−	−	−	−	−	−
D-Val			−	−	42.9	2.1	−	−	−	−
D-Met			−	−	−	−	−	−	−	−
D-Trp			−	−	−	−	−	−	−	−
D-Phe			N.D.^e^	−	10.7	0.8	N.D.	−	N.D.	−
D-*aIlo*-Ile			−	−	141.3	9.8	−	−	−	−
D-Leu			N.D.	−	194.0	5.9	N.D.	−	N.D.	−
D-Glu			118.7	4.3	54.7	1.7	161.0	5.7	65.5	2.0
D-Asn			11.9	2.5	10.9	2.4	14.0	3.0	4.9	0.8

^a^Details of the sample preparation are provided in the Methods.

^b^Concentrations cited are smaller one among the amino acid concentrations determined using NAC and NBC as chiral regents.

^c^Relative percentages of D-amino acids were calculated as 100 × [D-amino acid]/[D-amino acid + L-amino acid].

^d^−, not detected.

^e^N. D., not determined. The D-amino acid peak was confirmed, but the concentration of the D-amino acid was too low to be determined.

## Conclusion

In this study, we showed that differences in the D-amino acid contents of several kinds of vinegar reflect differences in both the sources and fermentation processes. In particular, we found that lactic fermentation is mainly responsible for the production of many of the D-amino acids present in tomato vinegar. Similar increases in total D-amino acid levels were seen in beer, wine and sake following inoculation and proliferation of lactic acid bacteria (Erbe and Brückner, 2000; Kato et al., 2011; Gogami et al., 2011). Although it is known that yeast and acetic acid bacteria also produce D-amino acids during the fermentation, this is the first report comparing the production of D-amino acids during alcoholic, acetic and lactic fermentation using the same lot of source, and it clarified the abundant contribution made by lactic fermentation to the production of D-amino acids. As it is well known, various lactic acid bacteria are involved in the production of many fermented foods, including cheese, yogurt, soy sauce, pickles and sauerkraut, and as a consequence, D-amino acids are present in many of these foods. The contribution made by D-amino acids to taste of foods has been expected, but whereas it remains largely unstudied. We would therefore suggest that the effects of the D-amino acids produced by lactic acid bacteria on the taste of fermented foods should be studied with the aim of applying D-amino acid to controls and improvements in the taste of fermented foods.

## Methods

### Materials

Yeast (*Saccharomyces cerevisiae*) and pectinase from *Aspergillus niger* were purchased from Oriental Yeast Co., Ltd. (Tokyo, Japan) and Shin Nihon Chemical Co., Ltd. (Anjyo, Japan), respectively. *N*-Acethyl-L-cysteine (NAC), *N*-*tert*-butyloxycarbonyl-L-cysteine (NBC) and *o*-phthaldialdehyde (OPA) were from Wako (Tokyo, Japan), Nova Biochemical (Waltham, MA, USA) and Nacalai Tesque (Kyoto, Japan), respectively. D-Amino acid oxidase from porcine kidney and catalase from bovine liver were purchased from Sigma (St. Louis, MO, USA). *Lactobacillus* and *Lactococcus* strains were from the Japan Collection of Microorganisms (JCM, Tsukuba, Japan).

### Vinegar samples

Eleven vinegars (Table 1) were provided by the Central Research Institute of Mizkan Group Corporation. These samples were stored at 4°C until use. In these 11 vinegars, three kinds of vinegar (high-brix nonglutinous brown rice black vinegar, high-brix nonglutinous brown rice black vinegar matured in barrel and high-brix apple vinegar) have the word “high-brix” in their names. The “high-brix” means that these vinegars were produced using a larger amount of initial material than were used in the production of vinegars lacking the “high-brix”. The initial material of “high-brix nonglutinous brown rice black vinegar” and “high-brix nonglutinous brown rice black vinegar matured in barrel” was nonglutinous brown rice, and one of “high-brix apple vinegar” was apple juice. High-brix nonglutinous brown rice black vinegar matured in barrel and lactic fermented tomato vinegar, which are not currently available on the market, served as test samples for new commodification. High-brix nonglutinous brown rice black vinegar matured in barrel was produced by the maturation of high-brix nonglutinous brown rice black vinegar at 25°C for a month in a 20-L barrel. On the other hand, the lactic fermented tomato vinegar was produced through acetic fermentation using the mixture of alcoholic fermented tomato juice and lactic fermented juice.

### D-Amino acids in five different samples collected during the manufacture of lactic fermented tomato vinegar

D-Amino acids in the following five samples produced by Mizkan Group Corporation (Figure 1) were analyzed: sample 1 was tomato juice that served as the source for other samples; sample 2 was alcoholic fermented tomato juice; sample 3 was acetic fermented tomato juice; sample 4 was tomato juice before lactic fermentation; and sample 5 was lactic fermented tomato juice. As shown in Figure 1, these five samples were made on 3-L volumes from a single lot of tomatoes through two separate lines. Sample 1 (tomato juice-source) was prepared by mixing crushed tomatoes with water (2.5 kg-tomato/L). For the preparation of sample 2, the tomato juice-source was incubated at 28°C for 96 h after the addition of 0.5% (g/v) yeast and 0.3% (g/v) pectinase (alcoholic fermentation). After alcoholic fermentation, the tomato juice was filtered through filter paper (Advantec grade No. 2 qualitative filter paper; Advantec, Tokyo) and diatomaceous earth, and then heat-treated at 90°C for 1 min. For the preparation of sample 3, acetic acid bacteria (on the order of 10^5^ cells) were inoculated into the sample 2 (3 L), after which acetic fermentation was proceeded at 32°C for 110 h. The resultant acetic fermented tomato juice was heat-treated and filtered as described above (sample 3). Sample 4 was produced by the addition of 0.3% pectinase to the sample 1 (3 L), followed by incubation at 60°C for 1 h and heat-treatment at 92°C for 30 min. To produce sample 5, lactic acid bacteria (10^5^ cells) were added to sample 4 (3 L), and the mixture was fermented at 30°C for 90 h. The lactic fermented tomato juice was then subjected to heat-treatment at 79°C for 10 min and filtered as described above (sample 5).

### D-Amino acids in culture media conditioned by lactic acid bacteria

D-Amino acids were analyzed in the culture medium of eight lactic acid bacterial strains (Table 5). These strains were cultured to the stationary phase in 5 mL of MRS medium (Becton, Dickinson and Co., Franklin Lakes, NJ, USA) at their respective optimum cultivation temperatures (Table 5). An aliquot (1 mL) of the culture medium was then centrifuged (10,000 × *g* for 1 min at 4°C), and the supernatant was filtered through polytetrafluoroethylene membrane filters (4-mm diameter, 0.2-μm pore size; Merck Millipore, Darmstadt, Germany). The prepared samples were stored at −20°C until use.

### Sample preparation for amino acid analyses

To prepare samples for determination of free D- and L-amino acids, an aliquot of each sample (500 μL) was initially neutralized with 10 M NaOH and diluted to 600 μL with purified water. This solution was then filtered through an Amicon Ultra 0.5 mL centrifugal filter 3 K device (Merck Millipore) with centrifugation (14,000 × *g* for 15 min at 4°C).

### Derivatization of amino acids

Amino acids were derivatized using OPA plus NAC or NBC (Aswad, 1984; Hashimoto et al., 1992). Two methanolic solutions containing derivatizing reagents were prepared. Methanolic solution A was prepared by dissolving 8 mg of OPA and 10 mg of NAC in 1 mL of methanol, while methanolic solution B was prepared by dissolving 10 mg of OPA and 10 mg of NBC in 1 mL of methanol. The reaction mixture (250 μL) for the derivatization consisted of 25 μL of sample, 50 μL of methanolic solution (A or B), and 175 μL of 0.4 M borate-NaOH buffer (pH 10.4). After derivatization for 2 min at room temperature in the dark, an aliquot (1 μL) of the reaction mixture was introduced into an ultra-performance liquid chromatography (UPLC) system (Waters, Milford, MA, USA).

### Standard solution

The standard solution consisted of 32 kinds of amino acids: D- and L-forms of Asp, Glu, Ser, Ala, Leu, Phe, Val, Met, tryptophan (Trp), Arg, histidine (His), threonine (Thr), Asn, tyrosine (Tyr) and glutamine (Gln), plus L-isoleucine (L-Ile) and D-*allo*-Ile. For the calibration curves, the standard solution was diluted to 7 concentrations (5, 25, 50, 100, 250, 500 and 1,000 μM) in 0.05 M HCl, after which the solutions were filtered through polytetrafluoroethylene membrane filters (4-mm diameter, 0.2-μm pore size, Merck Millipore).

### Operation of the UPLC system

The diastereoisomeric derivatives formed with OPA-NAC or OPA-NBC were applied to a Waters AccQ-Tag Ultra 2.1 × 100 mm column (Waters) in an the ACQUITY UPLC TUV system consisting of a Waters Binary Solvent Manager, a Waters Sample Manager, and a Waters FLR Detector. The excitation and emission wavelengths for fluorescent detection of amino acids were 350 nm and 450 nm, respectively. The data were processed using Empower 2 (Waters). The system was operated with a flow rate of 0.25 mL/min at 30°C. The UPLC gradient system for analysis of OPA-NAC derivatives (A = 50 mM sodium acetate, pH 5.9, and B = methanol) was 10 − 20% B over 3.2 min, 20% B for 1 min, 20 − 40% B over 3.6 min, 40% B for 1.2 min, 40 − 60% B over 3.8 min, 60% B for 1 min, and 60 − 10% B over 0.01 min. The gradient system used for analysis of OPA-NBC derivatives (A = 50 mM sodium acetate, pH 5.9, and B = acetonitrile) was 15 − 21% B over 7 min, 21–27.5% B over 1.5 min, 27.5% B for 2 min, 27.5 − 30% B over 1 min, 30 − 40% B over 2 min, 40% B for 0.5 min, and 40 − 15% B over 0.01 min.

### Analysis and quantification of D-amino acids

With the UPLC method, the peak heights were used for quantification of amino acids. In addition, D-amino acid peaks were identified based on their retention times, as verified by comparisons with authentic samples. Food samples usually contain any unidentified amino compounds that give the same retention time-peak as a D-amino acid. Therefore, the peaks of D-amino acids, except for D-Glu, D-Asp, D-Gln and D-Asn, were ascertained from the reduction in fluorescence intensity elicited by treatment with D-amino acid oxidase (DAO) from *Sus scrofa* (Tosa et al., 1974). This DAO has very broad substrate specificity, oxidizing 12 different D-amino acids, though not D-Glu, D-Asp, D-Gln or D-Asn (D’Aniello et al., 1993). Treatment with DAO removed the 12 susceptible D-amino acids from the food samples, causing the respective peaks to be smaller than those obtained with corresponding untreated samples. To treat samples with DAO, an aliquot of sample solution (200 μL) was mixed with 300 μL of 150 mM sodium pyrophosphate buffer (pH 8.5) containing 182.5 μg of DAO, 67.5 μg of catalase from bovine liver and 2 mM flavin adenine dinucleotide, and incubated for 12 h at 25°C. The sample was then subjected to UPLC analysis after the removal of DAO and catalase by filtration using an Amicon Ultra 0.5 mL centrifugal filter 3 K device (Merck Millipore).

Calibration curves were constructed by plotting peak heights against amino acid concentrations at ranging from 5 to 1,000 μM. For all 16 amino acid types tested, the relation was linear with regression coefficients above 0.999.

When amino acids were derivatized with OPA-NAC or OPA-NBC, any compounds containing an amino group in addition to D- and L-amino acids can also be modified. To decrease the effects of amino compounds other than α-amino acids when assaying D- and L-amino acids, the lower concentrations of amino acids were selected and assessed using in two analyses using NAC and NBC as chiral regents. D-His, L-Arg, D-Trp, and D-*allo*-Ile were detected using only when OPA-NAC was used as the chiral reagent, whereas L-Glu, D-Glu, L-Asn and D-Asn were detected only using OPA-NBC.

## Abbreviations

NAC: *N*-acethyl-L-cysteine
NBC: *N*-*tert*-butyloxycarbonyl-L-cysteine
OPA: *O*-phthaldialdehyde
UPLC: Ultra-performance liquid chromatography
DAO: D-amino acid oxidase.
